# In vivo selection of sfGFP variants with improved and reliable functionality in industrially important thermophilic bacteria

**DOI:** 10.1186/s13068-017-1008-5

**Published:** 2018-01-17

**Authors:** Elrike Frenzel, Jelmer Legebeke, Atze van Stralen, Richard van Kranenburg, Oscar P. Kuipers

**Affiliations:** 1 Department of Molecular Genetics, Groningen Biomolecular Sciences and Biotechnology Institute, Centre for Synthetic Biology, University of Groningen, Nijenborgh 7, 9747 AG Groningen, The Netherlands; 2 Laboratory of Microbiology, Wageningen University, Stippeneng 4, 6708 WE Wageningen, The Netherlands; 3 Corbion, Arkselsedijk 46, 4206 AC Gorinchem, The Netherlands

**Keywords:** GFP, sfGFP, Thermostability, Biotechnology, *Parageobacillus* sp., *Geobacillus* sp., Thermophilic bacteria, FACS, Protein engineering, In vivo application, Cyan, Yellow

## Abstract

**Background:**

Fluorescent reporter proteins (FP) have become an indispensable tool for the optimization of microbial cell factories and in synthetic biology per se. The applicability of the currently available FPs is, however, constrained by species-dependent performance and misfolding at elevated temperatures. To obtain functional reporters for thermophilic, biotechnologically important bacteria such as *Parageobacillus thermoglucosidasius*, an in vivo screening approach based on a mutational library of superfolder GFP was applied.

**Results:**

Flow cytometry-based benchmarking of a set of GFPs, sfGFPs and species-specific codon-optimized variants revealed that none of the proteins was satisfyingly detectable in *P. thermoglucosidasius* at its optimal growth temperature of 60 °C. An undirected mutagenesis approach coupled to fluorescence-activated cell sorting allowed the isolation of sfGFP variants that were extremely well expressed in the chassis background at 60 °C. Notably, a few nucleotide substitutions, including silent mutations, significantly improved the functionality and brightness. The best mutant sfGFP(N39D/A179A) showed an 885-fold enhanced mean fluorescence intensity (MFI) at 60 °C and is the most reliable reporter protein with respect to cell-to-cell variation and signal intensity reported so far. The in vitro spectral and thermostability properties were unaltered as compared to the parental sfGFP protein, strongly indicating that the combination of the amino acid exchange and an altered translation or folding speed, or protection from degradation, contribute to the strongly improved in vivo performance. Furthermore, sfGFP(N39D/A179A) and the newly developed cyan and yellow derivatives were successfully used for labeling several industrially relevant thermophilic bacilli, thus proving their broad applicability.

**Conclusions:**

This study illustrates the power of in vivo isolation of thermostable proteins to obtain reporters for highly efficient fluorescence labeling. Successful expression in a variety of thermophilic bacteria proved that the novel FPs are highly suitable for imaging and flow cytometry-based studies. This enables a reliable cell tracking and single-cell-based real-time monitoring of biological processes that are of industrial and biotechnological interest.

**Electronic supplementary material:**

The online version of this article (10.1186/s13068-017-1008-5) contains supplementary material, which is available to authorized users.

## Background

Among other thermophilic bacteria, the Gram-positive *Parageobacillus* and *Geobacillus* species receive increasing interest as platform organisms for industrial biotechnology. Besides their exploitation for the identification and production of novel thermostable enzymes [1–4], these species are nowadays considered important chassis to build microbial cell factories for bioconversion and consolidated bioprocessing, particularly in biorefining applications based on renewable resources such as plant biomass [5, 6]. Anticipated advantages of thermophilic organisms over mesophilic organisms are that their growth temperatures between 50 and 70 °C reduce the risk of contamination by mesophilic bacteria during fermentation processes, their reduced cooling costs, and the increased reaction rates at thermophilic temperatures. Additionally, the versatility of producing polysaccharide-degrading enzymes and their ability to use many different sugars make them attractive chassis organisms [7–12].

*Parageobacillus thermoglucosidasius*, which was originally described as *Bacillus thermoglucosidasius* [13] and *Geobacillus thermoglucosidasius* emended *thermoglucosidans* [14, 15], was recently taxonomically revised from the genus *Geobacillus* as monophyletic clade II organism [16]. This bacterium is of particular interest because it is a facultative anaerobe, with a mixed acid fermentation metabolism leading to the production of chemical building blocks such as lactate, formate, acetate, ethanol, and succinate.

In contrast to model organisms such as *Bacillus subtilis*, the development and application of tools for genetic manipulation of (*Para*)*geobacillus* species is still in its infancy. Lately, the improvement of transformation procedures [7, 17, 18] paved the way for directed strain engineering. Though limited by the requirement of heat stability and reliable expression at high temperatures, an initial set of important tools has been established. This includes temperature-stable and temperature-sensitive cloning and integration vectors, antibiotic resistance marker genes (reviewed in [19, 20]), natural, synthetic and inducible promoters and RBS [7, 21–23], and gene knock-out/knock-in systems [7, 24]. Among these tools, reporter genes with an easy and highly sensitive readout mode such as fluorescent proteins (FP) are of special interest. Their biotechnological applicability is broad, ranging from biosensors to output elements to assess the strength of promoter elements, which allows monitoring of flux changes or the redirection of metabolic fluxes during production processes [25, 26].

The first described and thoroughly analyzed FP is the 27-kDa green fluorescent protein (GFP), composed of 238 amino acids, which was isolated from the jellyfish *Aequorea victoria* [27, 28]. Its intrinsic fluorescence with an excitation maximum of 395 nm and an emission maximum at 509 nm emanates from a chromophore that resides in a kinked alpha-helix, surrounded by a beta-barrel structure. The beta-can is composed of 11 strongly interacting beta-strands and protects the inner microenvironment with flexible loops and lids (reviewed in [29]). The side chains of amino acids that are facing inside the barrel and interact with the chromophore, determine not only the spectroscopic features but also the structural stability (reviewed in [30]). The beta-barrel structure is folded before the chromophore itself is formed by autocatalytic cyclisation, dehydration and oxidation steps of the tripeptide Ser65–Tyr66–Gly67 [29]. Thus, fluorescence reflects a properly folded scaffold and a mature chromophore and is, therefore, a measure for the proper folding state of the protein in vitro and in vivo.

Due to their versatile applicability, a palette of more than 40 different FP variants is available nowadays [31]. These proteins have either been generated from the *A. victoria* GFP or were isolated from different species such as corals and anthozoa and further optimized for expression in eukaryotes and/or prokaryotes. These range from blue to far-red color varieties to pH and redox sensors, Ca^2+^-detectors, photoswitchable and timer proteins and many more, and are applied dependent on the target of research, the host organism and the compatibility of excitation and emission maxima to fluorescence detection techniques (for reviews, see [32–34]). Heterologous expression of FPs, however, has certain limitations. Previous studies showed that the performance is species dependent [35], which is presumed to be primarily based on the codon usage frequency of the respective organism. Moreover, especially the factor temperature has profound effects on FP performance in vivo, since the majority of FPs was derived from eukaryotes that thrive in colder habitats. It was independently reported that, despite successful construction of GFP reporter strains, fluorescence signals could not be detected in thermophilic pro- and eukaryotes grown above 45 °C [36, 37]. The temperature sensitivity of wild-type GFP is restricted to the folding process, since low-temperature exposed and properly folded proteins remain fluorescent to at least 65 °C [29]. Consequently, a fast and robust folding GFP derivative was developed and termed ‘superfolder’ sfGFP [38]. The introduction of folding and solubility-enhancing mutations yielded a protein that folds well even when fused to poorly folded polypeptides, has increased in vitro thermal stability and shows superior resistance against chemical denaturants in comparison to conventional GFPs [38]. Additional attempts were made to obtain thermostable mutants by rational engineering of GFP [39]. The characteristics of these variants were only tested with the purified proteins in in vitro assays, which do not provide insights into their in vivo functionality. A few recent studies reported on the in vivo use of sfGFP variants as output in thermophilic *Geobacillus* species [22, 23, 40]. However, the efficiency of FP expression at the single-cell level and thus the dynamic range of expression were not studied. To perform meaningful quantification of promoter activity changes in single cells or cell populations, it is of importance that the fluorescence intensity of a biomarker remains constant and has narrow signal amplitude.

To develop a reporter protein suitable for the expression in Gram-positive thermophilic hosts, we first benchmarked the performance of a set of FPs in *P. thermoglucosidasius* DSM 2542 by flow cytometry. We further employed a random mutagenesis approach of the best performing FP variant coupled to fluorescence-activated cell sorting for isolating brightly expressed mutants in the chassis background at 60 °C. This in vivo selection approach resulted in the identification of several thermostable variants. One highly thermostable version with improved brightness, sfGFP(N39D/A179A), was further engineered to generate color variants as well as analyzed for its suitability of usage in additional thermophilic species.

## Methods

### Bacterial strains and growth media

The bacterial strains used in this study are listed in Table 1. *E. coli* was routinely grown in lysogeny broth (LB) containing 10 g/L tryptone (Oxoid), 5 g/L NaCl and 5 g/L yeast extract (Carl Roth GmbH) at 37 °C. Plates were prepared with 15 g/L of agar. When required, the following antibiotics were added: chloramphenicol (15 µg/mL), ampicillin (100 µg/mL), and kanamycin (50 µg/mL).

**Table 1:** Bacterial strains used in this study

Bacterial strains	Relevant genotype	Source/reference
*Escherichia coli* Top10	General cloning host	Invitrogen
*Escherichia coli* BL21(DE3)	Protein expression host	MolGen strain collection
*Bacillus smithii* DSM 4216	Wild-type	DSMZ, Germany
*Bacillus coagulans* DSM 1	Wild-type	DSMZ, Germany
*Bacillus methanolicus* DSM 16454	Wild-type	DSMZ, Germany
*Parageobacillus thermoglucosidasius* DSM 2542	Wild-type	DSMZ, Germany
*Geobacillus thermodenitrificans* DSM 465	Wild-type	DSMZ, Germany
*Geobacillus thermodenitrificans* T12	Wild-type	[80]

*B. smithii* was grown in LB2 broth, pH 7.0 [41], consisting of 10 g/L tryptone (Becton–Dickinson), 5 g/L yeast extract (Carl Roth GmbH), and 100 mL/L 10× ESS (2.3 g/L K_2_HPO_4_, 5.1 g/L NH_4_Cl, 50.0 g/L NaCl, 14.7 g/L Na_2_SO_4_, 0.8 NaHCO_3_, 2.5 g/L KCl, 18.7 g/L MgCl_2_·6H_2_O, 4.1 g/L CaCl_2_·2H_2_O). *B. coagulans* was cultivated in BC broth, pH 6.5 [42], containing 10 g/L glycine, 10 g/L yeast extract (Carl Roth GmbH), 10 g/L Bis–Tris buffer, 2 g/L (NH_4_)_2_HPO_4_, 3.5 g/L (NH_4_)_2_SO_4_, and 1 mL/L trace elements solution (5 g/L MgCl_2_, 3 g/L CaCl_2_, 0.05 g/L ZnCl_2_, 0.03 g/L MnCl_2_·4H_2_O, 0.3 g/L H_2_BO, 0.2 g/L CoCl_2_·6H_2_O, 0.01 g/L CuCl_2_·2H_2_O, 0.02 g/L NiSO_4_·6H_2_O, 0.03 g/L Na_2_MoO_4_·2H_2_O). *B. methanolicus* was grown in BM medium (DSMZ Medium No. 1192) at pH 7.0 (37.4 g/L marine broth (Difco), 0.02 g/L biotin, 0.001 g/L vitamin B_12_, and 4 mL/L methanol). *P. thermoglucosidasius* was cultivated in TGP broth [21], pH 7.0 (17 g/L tryptone (Oxoid), 3 g/L soy peptone (Oxoid), 5 g/L NaCl, 2.5 g/L K_2_HPO_4_, and post-autoclaved addition of filter-sterilized pyruvate and glycerol to final concentrations of 4 g/L and 4 mL/L). *G. thermodenitrificans* was grown in LB2D broth, pH 7.0 (10 g/L tryptone (Becton–Dickinson), 10 g/L NaCl, 5 g/L yeast extract (Carl Roth GmbH), 100 mL/L 10× ESS (2.5 g/L K_2_HPO_4_, 10 g/L NH_4_Cl, 30 g/L NaCl, 15 g/L Na_2_SO_4_, 0.8 g/L NaHCO_3_, 10 g/L KCl, 18 g/L MgCl_2_ 6H_2_O, 3 g/L CaCl_2_ 2H_2_O).

If not stated otherwise, cells were first pre-grown from glycerol stocks for 16 h in their respective growth medium and then 1:100 diluted into fresh medium in a 1/10th volume of the growth flasks. To grow the strains on plates, above-stated broths were solidified with 5 g/L gelrite (Carl Roth GmbH).

### Transformation procedures for thermophilic bacteria

One hundred milliliters of pre-warmed, strain-specific broth was inoculated from pre-cultures as follows: LB2 was inoculated with *B. smithii* to an OD600 of 0.08 and shaken at 55 °C, 130 rpm; BC broth was inoculated with *B. coagulans* at an OD600 of 0.10 and shaken at 45 °C, 120 rpm; SOBsuc broth was inoculated with *B. methanolicus* to an OD600 of 0.01 and shaken at 50 °C, 170 rpm; TGP was inoculated with *P. thermoglucosidasius* to an OD600 of 0.05 and shaken at 60 °C, 170 rpm; and LB2D was inoculated with *G. thermodenitrificans* to an OD600 of 0.1 and aerated at 55 °C, 140 rpm.

The cells were grown until the following optical densities were reached: *B. smithii* and *B. coagulans* OD600 of 0.5, *B. methanolicus* OD600 of 0.25, and *P. thermoglucosidasius* and *G. thermodenitrificans* OD600 of 1.0. The cells were centrifuged in pre-chilled 50-mL tubes: *B. smithii*: 15 min, 4700 rpm, 4 °C; *B. coagulans*: 10 min, 6000 rpm, 4 °C; *B. methanolicus* after 5 min on ice: 5 min, 3000×*g*, 4 °C; *P. thermoglucosidasius* after 10 min on ice: 10 min, 5000 rpm, 4 °C; and *G. thermodenitrificans* after 5 min on ice: 15 min, 4700 rpm, 4 °C. The cell pellets were resuspended in 50 mL SG buffer (171.2 g/L sucrose, 0.2 g/L MgCl_2_·H_2_O, 50 mL/L glycerol) for *B. smithii*; 50 mL EPB buffer (0.68 g/L KH_2_PO_4_, 72.8 g/L sorbitol, 0.8 g/L MgCl_2_, 100 mL/L glycerol, pH 4.5) for *B. coagulans*; 3.5 mL EP buffer (0.476 g/L HEPES, 250 g/L PEG 8000, pH 7.0) for *B. methanolicus*; 50 mL EPO buffer (91 g/L sorbitol and mannitol, 117.2 mL/L 85% glycerol) for *P. thermoglucosidasius*; and 50 mL EPO2 buffer (MilliQ plus 10% glycerol) for *G. thermodenitrificans*. Subsequently, cells were centrifuged and resuspended again in the following volumes: *B. smithii* 50, 25, and 12.5 mL SG buffer (4 °C, 15 min, 4700 rpm); *B. coagulans* 25 and 12.5 mL EPB buffer (4 °C, 10 min, 6000 rpm); *B. methanolicus* 3.5 mL EP buffer (4 °C, 5 min, 3000×*g*); *P. thermoglucosidasius* 25, 10, and 10 mL EPO buffer (4 °C, 10 min, 6000 rpm); and *G. thermodenitrificans* 50, 25, and 10 mL EPO2 buffer (4 °C, 15 min, 4700 rpm). The final pellets were resuspended and aliquots were stored at − 80 °C: *B. smithii* 240 µL SG buffer divided into 75 µL aliquots; *B. coagulans* 1 mL EPB buffer divided into 100 µL aliquots; *B. methanolicus* 200 µL EP buffer divided into 100 µL aliquots; *P. thermoglucosidasius* 750 µL EPO buffer divided into 65 µL aliquots; and *G. thermodenitrificans* 900 µL EPO2 buffer divided into 65 µL aliquots.

For electroporation, 1 µg of plasmid DNA was added to the aliquots of competent thermophilic bacteria. Different electroporation cuvette gap sizes were used: 0.1 cm for *B. smithii* and *P. thermoglucosidasius* and 0.2 cm for *G. thermodenitrificans*, *B. methanolicus*, *B. coagulans* and *G. stearothermophilus.* The following electroporation settings were used: *B. smithii* 1.5 kV, 25 μF, 600 Ω; *B. coagulans* 1.6 kV, 25 μF, 200 Ω; *B. methanolicus* and *G. thermodenitrificans* 2.5 kV, 25 μF, 200 Ω; and *P. thermoglucosidasius* 2.5 kV, 10 μF, 600 Ω.

After pulsing, cells were inoculated in 1 mL pre-warmed culture media and incubated under the following conditions: *B. smithii* 55 °C, 150 rpm, 3 h in ME2 broth; *B. coagulans* 45 °C, 160 rpm, 3 h in RM medium, *B. methanolicus* first 45 °C, 120 rpm, 2 h and then 50 °C, 200 rpm, 4 h in SOBsuc medium; *P. thermoglucosidasius* 52 °C, 140 rpm, 2 h in TGP broth, and *G. thermodenitrificans* 55 °C, 140 rpm, 2 h in LB2D medium. Afterwards, cells were plated and incubated on the following growth media: *B. smithii* at 55 °C on LB2 containing 7 μg/mL chloramphenicol; *B. coagulans* at 45 °C on BC containing 5 μg/mL chloramphenicol *B. methanolicus* at 50 °C on BM with 5 μg/mL chloramphenicol; *P. thermoglucosidasius* at 52 °C on TGP with 10 μg/mL chloramphenicol; *G. thermodenitrificans* at 55 °C on LB2 with 5 μg/mL chloramphenicol.

### Recombinant DNA techniques

DNA isolation, manipulation and transformation of *E. coli* were carried out according to standard procedures [43]. All enzymes were obtained from Thermo Fisher Scientific. Phusion High-Fidelity DNA polymerase was used for cloning and sequencing purposes and the DreamTaq polymerase for colony PCR. Plasmid constructs were verified by double-strand DNA sequencing (Macrogen).

### Colony PCR of thermophilic bacteria

Colonies were resuspended in 200 µL MilliQ water, vortexed and centrifuged for 2 min at 12,000 rpm. To the cell pellet, 100 µL InstaGene Matrix (Bio-Rad) was added and the samples were incubated for 30 min at 55 °C. After vortexing, the cells were lysed by incubation at 100 °C for 8 min and the cell debris was removed by centrifugation (3 min, 13,000 rpm). The DNA-containing supernatant was subsequently used for PCR with plasmid-specific oligos pNW33N_for and pNW33N_rev (for oligonucleotides used in this study, see Additional file 1: Table S1).

### Construction of FP expression plasmids

All oligonucleotides and plasmids used in this study are listed in Additional files 1 and 2: Tables S1, S2, respectively. To generate the *E. coli*–*Geobacillus* shuttle vector pNW-Ppta-3TER, the *pta* promoter was amplified with the oligonucleotides Ppta_for and Ppta_rev from genomic DNA of *P. thermoglucosidasius* DSM 2542. The fragment was cut with EcoRI and SmaI and cloned into the equally cut pNW33N backbone. The threefold terminator was amplified from plasmid pKB01-sfGFP(Sp) using the oligonucleotide pair 3TER_for and 3TER_rev, cut with *Sph*I and *Hin*dIII and cloned into the *Sph*I and *Hin*dII digested plasmid pNW-P_pta_. The pNW-P_pta_-FP-3TER vectors containing FPs (Additional file 2) were constructed by amplification of the respective GFP genes while incorporating a 5′ end *Xba*I site and a 3′ end *Sph*I site: the *gfpmut3A* gene was amplified with primers GFPmut3A_for and GFPmut3A_rev from pAD123; *gfpuv* was amplified with primers GFPuv_for and GFPuv_rev from pSG1156; sfGFP_Gst_F and sfGFP_Gst_R were used for amplification of *sfGFP(Gst)* from PRHIII-sfGFP-pNW33N; and *gfp*+, *gfp(Sp)*, *sfgfp(Bs)*, *sfgfp(Sp)* and *sfgfp(iGEM)* were amplified with the primer pair pKB01_FP_for and pKB01_FP_rev from the respective plasmids of the pKB01 series (Additional file 2: Table S2). After restriction with *Xba*I and *Sph*I, PCR fragments were ligated into the *Xba*I and *Sph*I cut pNW-P_pta_-FP-3TER backbone.

Color variants of the thermostable sfGFPS70 protein were engineered by site-directed mutagenesis PCR. To introduce the cyan Y66W mutation, the 5′ end of sfGFP(N39D/A179A) was amplified with the primer pair sfGFP_Xba_F and sfGFP_Y66W_R and the 3′ end was amplified with sfGFP_Y66W_F and sfGFP_Sph_R (Additional file 1: Table S1). Purified PCR fragments were mixed in equimolar amounts and used as a template in a PCR using the primers sfGFP_Xba_F and sfGFP_Sph_R. For introducing the yellow T203Y mutation, the primer pairs sfGFP_Xba_F/sfGFP_T203Y_R and sfGFP_T203Y_F/sfGFP_Sph_R were used. The final amplification products were cut with *Xba*I and *Sph*I and cloned into pNW-P_pta_-3TER, which was cut with the same enzymes, giving rise to pNW-sfCFPS102 and pNW-sfYFPS102.

**Table 2:** Properties of GFP derivatives used in this study for benchmarking the in vivo expression in *P. thermoglucosidasius* DSM 2542

GFP	Changes to *A. victoria* wild-type GFP	Properties	Gene/codon optimization method	References
GFPuv *aka* “cycle3” mutant	F99S, M153T, V163A	Improved brightness by excitation with UV light	Obtained by three cycles of DNA shuffling	[81]
GFP+	F64L, S65T, Q80R, F99S, M153T, V163A	320-fold improved brightness in comparison to wild-type GFP	Introduction of GFPmut1 mutation into GFPuv by PCR	[55]
GFPmut3A	S65G, S72A	FACS-optimized mutant	Obtained by random mutagenesis and cell sorting by FACS	[82]
GFP(Sp)	M1MV, S65A, V68L, S72A, A206K	Codon-optimized	Codon optimization for *S. pneumoniae* using OptimumGene software	[83]
sfGFP	S30R, Y39N, F64L, S65T, Q80R, F99S, N105T, Y145F, M153T, V163A, I171V, A206V	Original superfolder GFP	Obtained by DNA shuffling and fusion to poorly folding proteins. Based on the “folding reporter” protein that combined cycle3 (F99S, M153T, V163A) and enhanced GFP mutations (F64L, S65T)	[38]
sfGFP(Gst)	S30R, Y39N, F64L, S65T, Q80R, F99S, N105T, Y145F, M153T, V163A, I171V, A206V	Codon-optimized superfolder GFP	Codon optimization for *Geobacillus stearothermophilus* performed by GeneArt	[40]
sfGFP(iGEM)	S30R, Y39N, F64L, S65T, S72A, F99S, N105T, Y145F, M153T, V163A, I171V, A206V	Codon-optimized superfolder GFP, additional GFPmut3* mutation (S2R)	Codon optimization for *E. coli* and *B. subtilis* performed by GeneArt	[84]
sfGFP(Bs)	S30R, Y39N, F64L, S65T, Q80R, F99S, N105T, Y145F, M153T, V163A, I171V, A206V	Codon-optimized superfolder GFP	*B. subtilis* using dual codon method	[35]
sfGFP(Sp)	S30R, Y39N, F64L, S65T, Q80R, F99S, N105T, Y145F, M153T, V163A, I171V, A206V	Codon-optimized superfolder GFP	*S. pneumoniae* using OPTIMIZER program	[35]

For expression in *E. coli* and in vitro analysis of the proteins, FPs were amplified with the primers LICv1sfGFP_Fs and LICv1sfGFP_Rs using the plasmids pNW-sfGFP(Sp), pNW-sfGFP(N39D/A179A), pNW-sfGCPS102 and pNW-sfYFPS102 as the template. Ligation-independent cloning into the plasmid pETHis6TEVLic (1B) was performed as described previously [44], thereby placing the FPs downstream of an IPTG-inducible promoter and of a TEV protease-cleavable His_6_ tag for affinity purification.

### Generation of sfGFP(Sp) mutational library

The sfGFP(Sp) gene was randomly mutagenized using the GeneMorph II Random Mutagenesis Kit (Agilent Genomics) according to the manufacturers’ instruction. The primers pKB01derMut_F and pKB01derMut_R were designed to contain a *Xba*I and a *Sph*I restriction site and to leave the start codon and the three stop codons of the *sfGFP(Sp)* gene intact, respectively. The amplified fragments were digested with *Xba*I and *Sph*I and ligated into pNW-P_pta_-3TER, downstream of the constitutive *pta* promoter of *P. thermoglucosidasius* DSM 2542. Ligation products were transformed by electroporation (25 µF, 200 Ω, 2.5 kV; 0.2-mm cuvettes) into 40 µL aliquots of electrocompetent *E. coli* Top10, yielding a final library size of 125,000 clones. To determine the mutation frequency, twenty randomly selected colonies were used for plasmid isolation and sequencing with the primers pNW33N_for and pNW33N_rev. All *E. coli* Top10 colonies were pooled by washing the colonies with 10 mL of LB-Cm15 medium from the transformation plates. Fifty milliliters of the cell suspension was pelleted (4 °C, 5 min, 6000*g*) and used for isolation of the plasmid pool with the Jetstar Plasmid Purification Midi kit (Genprice) for transformation of *P. thermoglucosidasius* DSM 2542.

### Isolation of temperature-stable GFP variants by fluorescence-activated cell sorting (FACS) of *P. thermoglucosidasius*

Before sorting, the sample line was sterilized with 75% EtOH for 5 min. Cells grown to mid-exponential growth phase (OD_600_ = 1.5–2.5) were diluted into sterile filtered, 65 °C-pre-warmed PBS buffer, pH 7.0, and instantly subjected to FACS with a FACS Aria II (Becton–Dickinson). Samples were sorted using a 70-µm nozzle choosing the highest purity setting (yield mask: 0, purity mask: 32, phase mask: 0). The population was gated by forward scatter (488/10 nm), side scatter (488/10 nm), and by the GFP emission (ex 525/50 nm, 505 nm LP filter) windows (all in log scale). Libraries grown at 60 °C were sorted with a narrow gate (GFP-A 10^4^ − 2 × 10^5^), thereby isolating clones from the 0.1% subgroup of highest fluorescence of population. Libraries grown at 65 °C were sorted with a broader gate (GFP-A 10^3^ – 2 × 10^5^), thereby isolating 2.0% of population corresponding of highly fluorescent clones. Approximately 100,000 clones were sorted into 5 mL of TGP medium containing 10 µg/mL chloramphenicol. From these, 100 µL were plated directly on TGP–Cm10 plates and incubated at 60 °C for subsequent analysis by colony fluorescence imaging. The remaining cells were used to inoculate 50 mL of fresh TGP–Cm10 medium and grown at 60 or 65 °C in 250-mL baffled flasks for 16 h. In total, two iterative rounds of sorting, outgrowth and repeated isolation of brightest clones were performed at 60 and 65 °C.

### Colony fluorescence imaging

Fluorescence signals of plate-grown colonies were captured using an Olympus MVX10 MacroZoom fluorescence microscope equipped with a PreciseExcite LED fluorescence illuminator (470 nm), a GFP filter set (ex 460/480 nm and em 495/540 nm), and an Olympus XM10 monochrome camera.

### Fluorescence microplate assay

DSM 2542 cultures were grown at 45–60 °C in TGP broth with 10 µg/mL chloramphenicol as described above. Every 30 min, 200 µL of cells were transferred to a microtiter plate and the fluorescence was recorded in the top-reading mode with an Infinite 200 plate reader (Tecan Group) equipped with a GFP filter set (ex 485/20 nm, em 535/25 nm). The GFP signals were corrected for OD600, background fluorescence of the broth, and for autofluorescence (wild-type cells) as previously described [35].

### Flow cytometry (FC) of *P. thermoglucosidasius*

Single-cell fluorescence measurements were made with a FACSCanto flow cytometer (BD Biosciences) with 488 nm excitation from a 20 mW solid-state laser. Geobacillus pre-cultures were obtained by inoculating 10 mL TGP supplemented with 8 μg/mL chloramphenicol from glycerol stocks and incubation at 60 °C and 170 rpm. After 16 h, cells were diluted to an OD600 of 0.08 into 25 mL pre-warmed, fresh TGP-Cm8 in 250-mL baffled flasks, and incubated at the desired temperatures at 170 rpm. Samples taken after specific time intervals (1, 2, 4, 6, 8, 10 and 12 h) or at an OD600 of 1.0 were immediately diluted (at least 1:100) with pre-warmed phosphate buffer solution (PBS, pH 7.0). Per sample, 100,000 events were recorded by measuring the fluorescence at 530 nm/30 nm band-pass width. The instrument settings were as follows: threshold-SSC 200, FCS 200, FL1 600 V, all in log amplification. The data were captured with the BD FACSdiva software (version 5.0.3) and for data analysis Flowing Software (version 2.5.1) and FCSalyzer (version 0.9.13) were used.

### Fluorescence microscopy

Strains containing the pNW-P_pta_-FP plasmids (Additional file 2) were grown for 16 h on solid media (*B. smithii* LB2 plates with 5 μg/mL chloramphenicol at 55 °C, *B. coagulans* BC plates with 9 μg/mL chloramphenicol and *B. methanolicus* BM plates with 5 μg/mL chloramphenicol at 45 °C, and *P. thermoglucosidasius* and *G. thermodenitrificans* on TGP plates with 8 μg/mL chloramphenicol at 60 °C). Cells were resuspended in PBS and transferred to a microscope slide coated with 2% PBS-agarose. Alternatively, cells from liquid cultures were spotted on the agarose slides. Images were taken using a Deltavision Elite microscope (Applied Precision) with a sCMOS camera and SSI Solid-State Illumination (Applied Precision) through a 100 × oil immersion objective (phase contrast) and 50 mW laser illumination for fluorescence combined with Quad (for GFP) or C/YFP/mCH (for CFP and YFP) polychroic filters. Excitation and emission filters were as follows: GFP, ex 475/28 nm and em 523/36 nm; CFP, ex 438/24 nm and em 475/28 nm; YFP, ex 513/17 nm and em 548/38 nm. Images were acquired with the softWoRx software 5.5 (Applied Precision) and processed with FIJI (https://fiji.sc/).

### Overexpression and purification of FPs from *E. coli*

FPs were expressed from the pETHis6TEVLic plasmids in *E. coli* BL21(DE3) (Additional file 2: Table S2). After 1 mM IPTG induction, cells were grown in LB for 3 h (37 °C, 220 rpm). Cells were harvested (6000*g*, 4 °C, 10 min) and washed with buffer A (50 mM Tris pH 7.6, 50 mM KCl, 1 mM DTT, 0.1 mM PMSF, pH 7.4). The cell pellet was resuspended in 10 mL of lysis buffer (50 mM NaH_2_PO_4_, 300 mM NaCl, and 10 mM imidazole, pH 7.4, supplemented with oComplete EASYpack EDTA free protease inhibitor). After sonification on ice (10 min, amplitude 75%, 15 s on, 10 s off), the cell debris was removed by centrifugation (4 °C, 50 min, 16,200 g) and soluble proteins were purified with a TEV protease-cleavable, N-terminal His6 tag as follows: the supernatant was loaded onto 5-mL Histrap FF columns (GE Healthcare Life Sciences) and the protein was eluted according to the manufacturer’s instruction with an imidazole gradient (solution A: 50 mM NaH_2_PO_4_, 300 mM NaCl, 10 mM imidazole; solution B: 50 mM NaH_2_PO_4_, 300 mM NaCl, 500 mM imidazole) using the NGC chromatography system (Bio-Rad).

The N-terminal His6-tag was cleaved off with the TEV protease (ProTEV Plus, Promega) according to manufacturer’s instructions. The His6-tag and uncleaved proteins still containing the His6-tag were removed with the HisLink Protein Purification Resin (Promega) according to the manufacturer’s protocol. For in vitro characterization of FP properties, proteins were dialyzed against PBS buffer, pH 7.4, and concentrated using 10kD-cutoff concentrator tubes (Pierce Protein Concentrator PES, Thermo Fisher Scientific). The concentration of purified FP proteins was estimated by measurement at 280 nm using a Nanodrop spectrophotometer (Thermo Fisher Scientific) and by a Bradford assay with Coomassie G-250 solution (Bio-Rad).

### Absorbance and emission spectra of FPs

To measure the absorbance and emission spectra, the purified FP variants were diluted to 20 µg/mL in a 20 mM Tris–HCl, pH 7.5, 100 mM NaCl buffer solution. Absorption spectra were recorded between 250 and 600 nm using 1 nm step size with a UV-1600 PC spectrophotometer (VWR) at 22 °C. Fluorescence measurements were performed at 22 °C on a SynergyMX microplate reader (BIOTEK) using white 96-well assay plates (3610, Costar) to which 75 µL per well was added. Emission spectra were measured between 300 and 700 nm with a 1 nm step size by exciting at 444 or 485 nm, respectively.

### In vitro thermal stability of FPs

FPs were diluted to 20 µg/mL in 50 µL of TNG buffer (100 mM Tris, pH 7.5, 100 mM NaCl, 10% glycerol) into 96-well PCR plates (iCycler IQ, Bio-Rad). Denaturation was monitored in a 7300 real-time PCR system (Applied Biosystems) at 488 nm excitation and 530 nm emission (FAM filter). The unfolding profile was resolved between 20 and 99 °C with a 30 s/ °C stepwise increase of the temperature.

## Results

### Benchmarking GFP performance in *P. thermoglucosidasius* DSM 2542

**Fig. 1:**
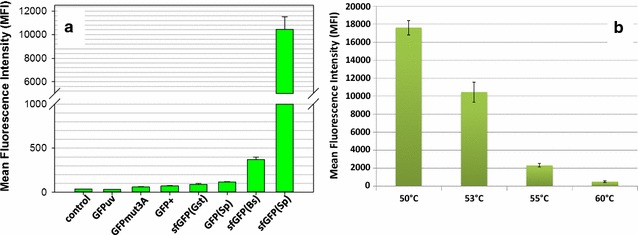
Benchmarking of GFP expression in *P. thermoglucosidasius* DSM 2542. **a** Expression of different GFP variants from pNW-Ppta-GFPx-3TER plasmids at 53 °C in TGP broth was analyzed by flow cytometry at the mid-exponential phase of growth. The mean fluorescence intensity (MFI) of 50,000 cells is shown. Control, autofluorescence of DSM 2542 containing a pNW-Ppta-3TER plasmids without *gfp* gene (**b**). Temperature-dependent performance of sfGFP(Sp) as assessed flow cytometry. The MFI of 50,000 cells at the mid-exponential phase of growth cells grown at indicated temperatures in TGP broth is presented. The averages of three biological triplicates are shown with error bars indicating standard deviations

To identify the most suitable GFP candidate for subsequent randomized mutagenesis and in vivo selection of temperature-stable mutants, we initially compared the expression of seven GFPs. These had been previously optimized for improved brightness, solubility, folding kinetics or were codon optimized for the application in different bacterial expression hosts (Table 2). The FP genes were inserted into the multiple cloning site of a derivative of the *E. coli*–*Bacillus* shuttle vector pNW33N (Additional file 2), thereby enabling a constitutive expression from the promoter of the *P. thermoglucosidasius*-derived housekeeping gene phosphate acetyltransferase (*pta*). The plasmid map is shown in Additional file 3. The transformed *P. thermoglucosidasius* DSM 2542 strains were grown at a moderate temperature of 53 °C in TGP broth and the distribution of fluorescence intensities of individual cells was evaluated from early logarithmic to late stationary phase using flow cytometry (exemplified in Additional file 3: Figure S1 B–D). We generally observed two trends: (i) cells transformed with less engineered GFP variants that are closely related to the originally isolated sequence of the *A. victoria* GFP showed either none or only marginal fluorescence above the autofluorescence background of *P. thermoglucosidasius* wild-type cells and (ii) cells transformed with sfGFP variants produced a detectable fluorescence signal (Fig. 1a). However, the signal intensities varied considerably amongst the sfGFP types, which differ in their nucleotide sequence, but not in their amino acid sequence (Table 2). The most surprising aspect was that a sfGFP variant, which had been codon optimized for expression in the closely related species *Geobacillus stearothermophilus* [40], gave very low detectable signals in the DSM 2542 background (Additional file 3: Figure S1 D). The sfGFP gene optimized for expression in *Streptococcus pneumonia* [sfGFP(Sp)], on the contrary, exhibited the highest mean fluorescence intensity (MFI) at 53 °C (Fig. 1a). When the strain was grown at the optimal growth temperature of 60 °C, the mean fluorescence decreased by 95% thereby indicating that sfGFP(Sp) was not functionally expressed, degraded or misfolded in a the majority of the cells (Fig. 1b). We, therefore, used the sfGFP(Sp) gene as starting material for an undirected mutagenesis approach to select the best performing mutants by in vivo screening.

### In vivo isolation of thermostable sfGFP variants

**Fig. 2:**
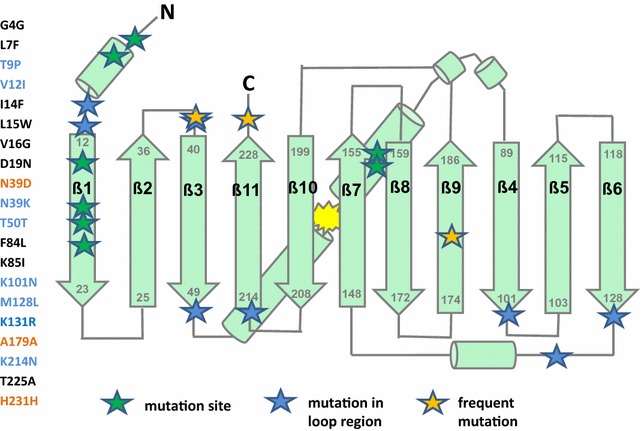
Topology map of sfGFP with thermostability-enhancing mutations isolated from in vivo FACS screening of DSM 2542. Left side: mutations found in the 50 sequenced mutants obtained by cell sorting from the sfGFP(Sp) library. Green stars: mutations found in at least 1 out of 50 protein sequences; blue stars: mutations that map to loop sites or flexible linker regions; orange stars: mutations that occurred in more than 10 out of 50 sfGFP(Sp) variants. Yellow cloud: chromophore

In an undirected, error-prone PCR approach, we randomly mutagenized the sfGFP(Sp) gene, thereby achieving a DNA library of 45,000 variants in *E. coli* with an average mutation rate of 2.8 exchanges per kb, which corresponds to an average of one to five amino acid exchanges per protein. After re-isolation from *E. coli*, the plasmid library was transformed into DSM 2542, which yielded a final library of ~ 6000 clones. Following four rounds of subsequent FACS enrichment of cells that produced the highest GFP emission signals at 55, 60 or 65 °C, respectively, (Additional file 4: Figure S2 A, B), the fluorescence intensity of 120 of the brightest individual colonies recovered on TGP plates was subsequently measured by flow cytometry (Additional file 4: Figure S2 C). Fifty variants showing the highest improvement in mean fluorescence intensity, in combination with the least cell-to-cell variation of GFP signal intensities, were further analyzed by sequencing. All variants were untruncated and contained a range of one to seven nucleotide substitutions per gene. Examples of sequences of isolated protein are provided in Additional file 5. Among the 20 mutations that were found in the 50 sequenced proteins, different trends could be observed: eight occurred in the flexible loop regions that connect the β-strands or constitute the lid of the β-barrel (Fig. 2). Other thermostability-promoting substitutions were found to be preferentially located in the N terminus and the first beta-strand. Four exchanges were synonymous, thereby leaving the amino acid sequence unchanged. Interestingly, A179A and H231H were reoccurring substitutions and accounted for 24 out of 50 and 10 out of 50 sequences, respectively. Moreover, these silent mutations replaced less favorable codons with codons that are more frequently used in *P. thermoglucosidasius*, with a ratio of 1.2 for A179A (GCT→GCC) and a ratio of 3.1 for H231H (CAC→CAT). An additional reoccurring mutation was the N39D variation found in 24 out of 50 variants.

### Characterization of the in vivo thermostability of mutant sfGFP(N39D/A179A)

**Fig. 3:**
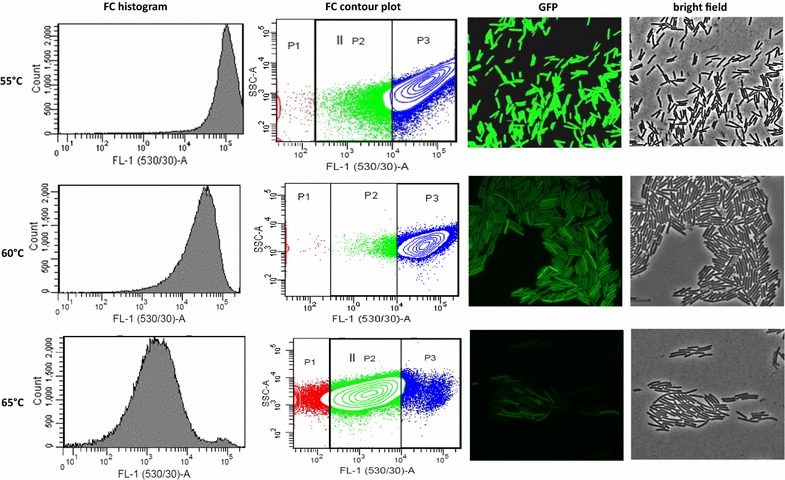
Visualization of sfGFP(N39D/A179A) expression in *P. thermoglucosidasius* DSM 2542 at different temperatures. Cells were grown to mid-exponential growth phase in TGP broth and the intensity of sfGFP(N39D/A179A) GFP emission in 50,000 single cells was analyzed by flow cytometry (FC). SSC-A denotes the side scatter area and equivalents the granularity of each detected cell; FL-1(530/20)-A denotes the fluorescence channel 1 area at 530/20 nm excitation, which equals the fluorescence intensity of each detected cell. The cells with fluorescence intensities within the range of the autofluorescence of wild-type DSM 2542 are colored red (gate P1). The cells with a fluorescence signal above the autofluorescence level of DSM 2542 are colored green (gate P2). Highly fluorescent cells are shown as blue dots in gate P3. The threshold between P2 and P3 was chosen arbitrarily. Fluorescence microscopy images were acquired at 100× magnification and at 475/28 nm excitation for 0.3 s and 523/36 nm emission. Representative FC histograms and microscopy images from three independent biological replicates are shown

Next, we analyzed one mutant displaying highly improved properties that contained two of the most frequently occurring mutations, N39D and A179A, in more detail (Table 3; Additional file 5). After re-cloning of sfGFP(N39D/A179A) into pNW-P_pta_-3TER and re-transformation into DSM 2542, this mutant still showed improved brightness compared to the parental sfGFP(Sp) reporter, indicating that the N39D and A179A mutations are crucial substitutions that affect its performance. The enhanced in vivo thermotolerance was further proven when the cells were subjected to increased temperatures (Fig. 3). In comparison to the original protein sfGFP(Sp), the MFI was increased 885-fold to 53,050 MFI at the optimal growth temperature of 60 °C. While exhibiting very high fluorescence signals at 55 °C that were at the detection limit for the flow cytometer (MFI of > 60,000), the variant lost activity at 65 °C (MFI = 8075) and displayed a broader heterogeneity in distribution of the signal intensity between single cells. Since 65 °C exceeds the optimum growth temperature and reflects a stress condition for DSM 2542, we hypothesized that the thermal stress might lead to a reduced folding efficiency or partial unfolding of sfGFP(N39D/A179A) proteins in a fraction of the cells.

**Table 3:** Properties of GFP derivatives used for setup of the mutational library and variants with improved in vivo expression in thermophilic bacilli

GFP	Changes to *A. victoria* wild-type GFP^a^	Properties	Optimization method	References
sfGFP(Sp)	S30R, Y39N, F64L, S65T, Q80R, F99S, N105T, Y145F, M153T, V163A, I171V, A206V	Used for random mutagenesis and FACS library construction in DSM 2542 in this study	*S. pneumoniae* using OPTIMIZER program	[35]
sfGFP(N39D/A179A)	S30R, *N39D*, F64L, S65T, Q80R, F99S, N105T, Y145F, M153T, V163A, I171V, *A179A*, A206V	Improved in vivo thermostability and brightness (885-fold) in comparison to sfGFP(Sp) at 60 °C	Random mutagenesis and cell sorting by FACS	This study
sfGFP(N39D/A179A/H231H)	S30R, *N39D*, F64L, S65T, Q80R, F99S, N105T, Y145F, M153T, V163A, I171V, *A179A*, A206V, *H231H*	Improved in vivo thermostability and brightness (310-fold) in comparison to sfGFP(Sp) at 60 °C	Directed mutagenesis by introduction of silent mutation H231H into sfGFP(N39D/A179A)	This study
sfGFP(N39D/Y66 W/A179A)	S30R, *N39D*, F64L, S65T, *Y66W*, Q80R, F99S, N105T, Y145F, M153T, V163A, I171V, *A179A*, A206V	Cyan variant with improved in vivo thermostability in thermophilic bacilli	Directed mutagenesis by introduction of cyan color mutation Y66 W into sfGFP(N39D/A179A)	This study
sfGFP(N39D/A179A/T203Y)	S30R, *N39D*, F64L, S65T, Q80R, F99S, N105T, Y145F, M153T, V163A, I171V, *A179A*, *T203Y*, A206V	Yellow variant with improved in vivo thermostability in thermophilic bacilli	Directed mutagenesis by introduction of yellow color mutation T203Y into sfGFP(N39D/A179A)	This study

^a^Italicized amino acid exchanges indicate changes to the sfGFP(Sp) protein

### Combination of the most frequently occurring thermostabilizing mutations does not lead to further improvement

**Fig. 4:**
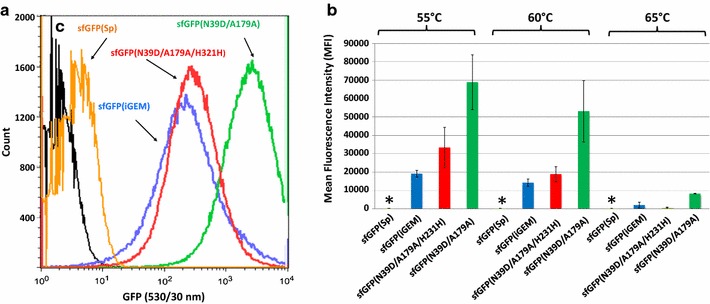
Influence of synonymous amino acid substitutions and N39D replacement on the in vivo performance of sfGFP in *P. thermoglucosidasius*. **a** Flow cytometry-based comparison of the signal intensities of mid-log DSM 2542 cells transformed with sfGFP(Sp), orange; sfGFP(iGEM), blue; sfGFP(N39D, A179A, H231H), red; sfGFP(N39D/A179A) (green) grown at 60 °C in TGP broth. C, control cells containing the pNW-Ppta-3TER plasmid without *gfp* gene (black). Representative histograms of three independent biological replicates are shown. **b** Analysis of temperature-dependent functionality of the four sfGFP variants at mid-exponential growth phase in DSM 2542 as assessed by flow cytometry. The MFI is presented in relative fluorescence units (R.F.U.) and is the average of three independent biological replicates with error bars showing standard deviations. Asterisks denote the detection of fluorescence signals marginally above the autofluorescence of mock cells as shown in control (c) in **a**

We next opted to study whether the combination of all three most frequently occurring mutations, N39D, A179A and H231H (Fig. 2; Table 3), would lead to synergistic effects in terms of improving the thermostability of the protein. The silent H231H mutation (CAC→CAT) was introduced via site-directed mutagenesis PCR into the sfGFP(N39D/A179A) variant. The resulting mutant, termed sfGFP(N39D/A179A/H231H), was expressed from pNW-P_pta_-sfGFP(N39D/A179A/H231H)-3TER in DSM 2452 under the same conditions as stated above. We additionally subcloned the sfGFP(iGEM) gene (Table 2), because its functional, although temperature-dependent expression in a *Geobacillus* strain had meanwhile been reported [22]. Comparison of signal intensities from cells grown at 60 °C revealed that i) the combination of thermostability-enhancing mutations led to reduced fluorescence of the sfGFP(N39D/A179A/H231H) variant and ii) next to reduced signal intensities, the sfGFP(iGEM) variant showed an extremely broad dynamic range when comparing the signal intensity derived from single cells. This indicates that this variant is not equally well expressed and/or folded at 60 °C, thus making it less suitable as a promoter output element in *P. thermoglucosidasius* DSM 2542 (Fig. 4a).

The effect was becoming more pronounced when the mean fluorescence intensities of the original protein, sfGFP(Sp), its derivatives sfGFP(N39D/A179A) and sfGFP(N39D/A179A/H231H) and the sfGFP(iGEM) variant were compared at 55, 60 and 65 °C, respectively (Fig. 4b). We, therefore, concluded that the nucleotide sequence and thus the codon usage must play an important role in conferring functional expression in dependence on the temperature. Additionally, the N39D amino acid exchange seems to further optimize the expression and/or the folding at higher temperatures, making it the most reliable variant with respect to cell-to-cell variation and signal intensity for (*Para*)*geobacilli* reported so far.

### In vivo functionality of sfGFP(N39D/A179A) in different host backgrounds

**Fig. 5:**
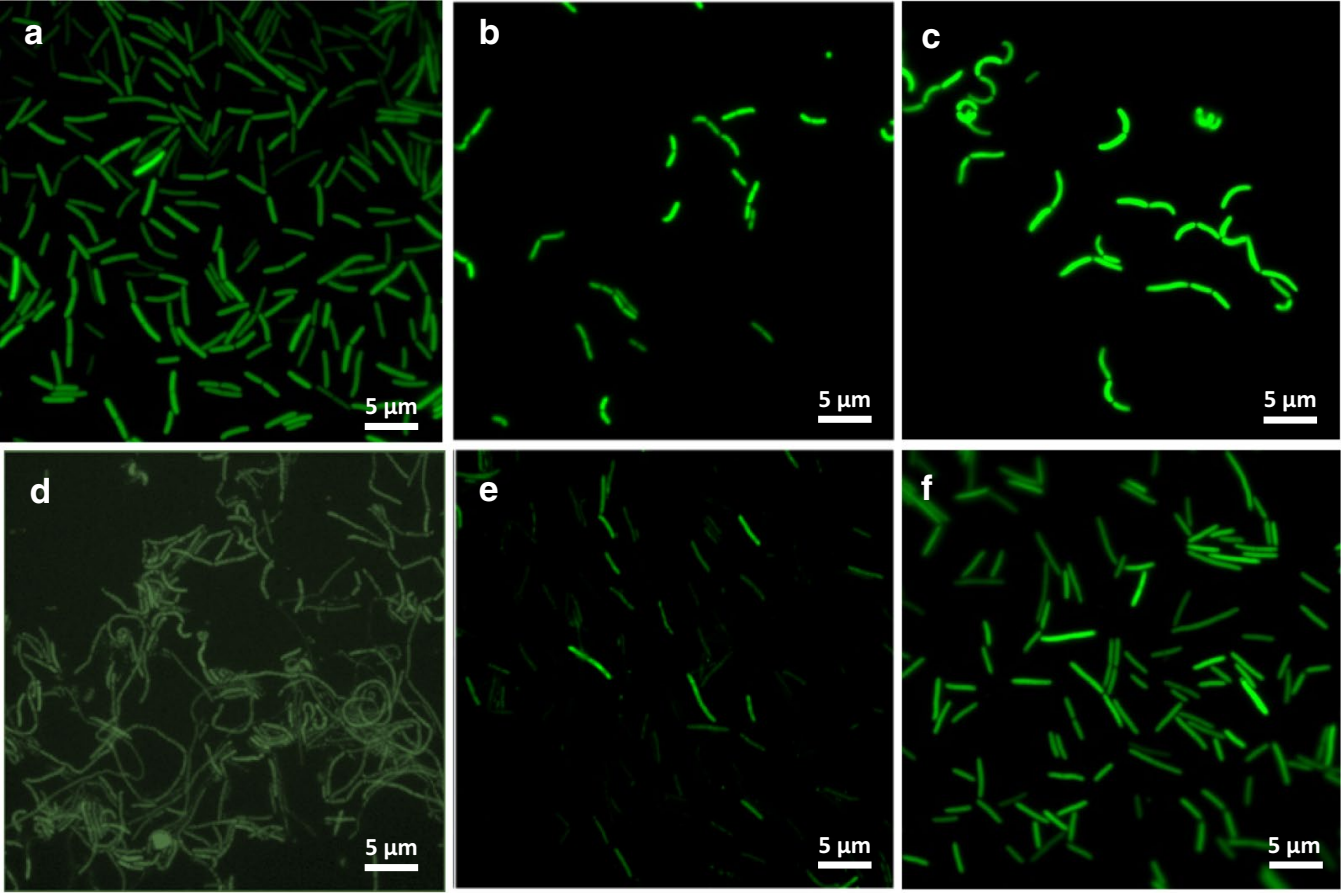
Visualization of sfGFP(N39D/A179A) expression in different thermophilic spore-forming species by fluorescence microscopy. Expression of the thermostable GFP variant sfGFP(N39D/A179A) from the plasmid pNW-Ppta-sfGFP(N39D/A179A)-3TER was imaged after growing the transformed strains on growth media as specified in “Methods”. **a** *P. thermoglucosidasius* DSM 2542 (60 °C), **b** *B. smithii* DSM 4216 (55 °C), **c** *B. coagulans* DSM 1 (50 °C), **d** *B. methanolicus* DSM 16454 (60 °C), **e** *G. thermodenitrificans* DSM 465 (60 °C), **f** *G. thermodenitrificans* T12 (60 °C). Excitation time, excitation and emission wavelengths and filter settings are given in “Methods”

Since there is a limited number of reports on the usage of GFP reporters in thermophilic spore formers, we next tested the applicability of the improved sfGFP(N39D/A179A) variant in a set of (moderately) thermophilic *Bacillus* and (*Para)geobacillus* strains, which are currently in the focus of establishment and expansion as industrial platform organisms (Table 1). Although the expression of the thermostable sfGFP(N39D/A179A) protein was driven by the *P. thermoglucosidasius*-derived constitutively active *pta* promoter from the plasmid pNW-P_pta_-sfGFP(N39D7A179A-3TER), all tested strains functionally expressed the reporter protein, as visualized by fluorescence microscopy (Fig. 5). This underpins that despite of deviating codon usage in these organisms, the in vivo-selected mutations confer an advantage for a reliable expression of our in vivo-isolated reporter protein at thermophilic temperatures.

### In vivo functionality of sfGFP(N39D/A179A) cyan and yellow derivatives in different host backgrounds

**Fig. 6:**
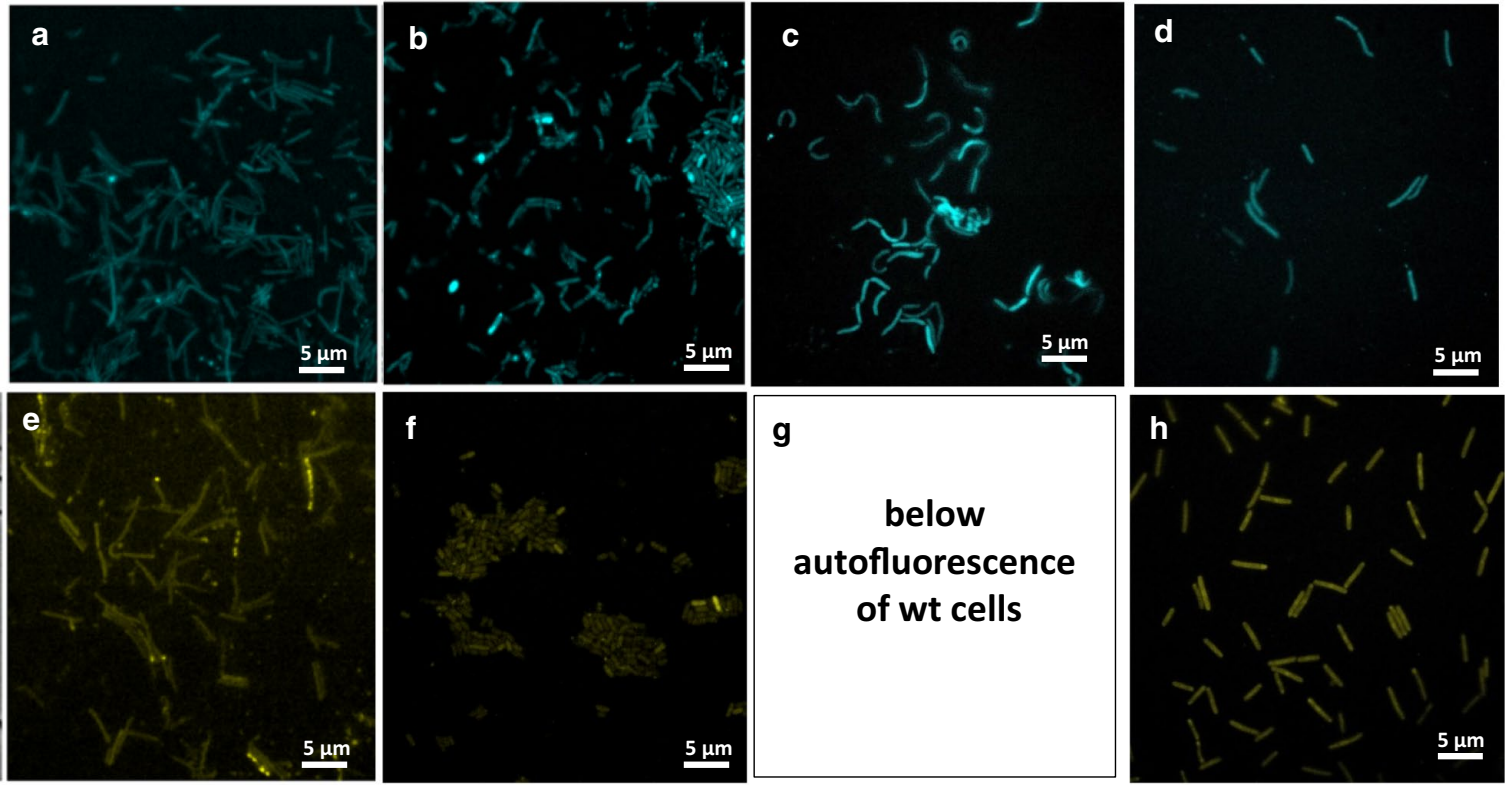
Visualization of expression of FP color variants in different (moderate) thermophilic spore-forming species. Expression of the cyan variant sfCFP(N39D/A179A) and the yellow variant sfYFP(N39D/A179A) from the corresponding pNW-P_pta_-GFPx-3TER plasmids was monitored after growing the transformed strains at their indicated optimal growth temperatures. **a**, **e** *P. thermoglucosidasius* DSM 2542 (60 °C), **b**, **f** *B. smithii* DSM 4216 (55 °C), **c**, **g** *B. coagulans* DSM 1 (50 °C), **d**, **h** *G. thermodenitrificans* T12 (60 °C). For excitation time, excitation and emission wavelengths and filter settings see “Methods”

For dual- or multi-labeling strategies, it would be desirable to have additional spectral FP variants that are thermostable. We thus introduced the mutations Y66W or T203Y that had previously been described to lead to cyan and yellow fluorescent color variants of GFP, respectively [45, 46] into sfGFP(N39D/A179A). The gene was mutated by site-directed mutagenesis PCR and the resulting sfCFP(N39D/A179A) and sfYFP(N39D/A179A) amplicons were cloned into the pNW-P_pta_-3TER plasmid. To analyze whether the newly introduced nucleotide exchanges impact the expression of the proteins in vivo, the plasmids were transformed into *P. thermoglucosidasius* DSM 2542 and into additional *Bacillus* and *Geobacillus* type strains and the expression was monitored by fluorescence microscopy (Fig. 6). A clearly detectable fluorescence signal illustrates that the applicability of the cyan and yellow color derivatives of sfGFP(N39D/A179A) was not restricted to *P. thermoglucosidasius*. While sfCFP(N39D/A179A and sfYFPS(N39D/A179A) were also functionally expressed in *B. smithii, B. coagulans* and *G. thermodenitrificans*, we were repeatedly unable to obtain transformants of *B. methanolicus* because of their low transformation efficiency. However, since we did not test the expression strength of the *pta* promoter in these hosts, it might be possible that stronger and/or species-specific promoters and plasmid backbones would lead to detectable fluorescence. The same applies to the detection of yellow fluorescence in *B. coagulans* DSM 1, which might be increased above the level of cellular autofluorescence when a stronger promoter would be used to drive sfYFP expression.

### In vitro properties of sfGFP(N39D/A179A) and its cyan and yellow derivatives

**Fig. 7:**
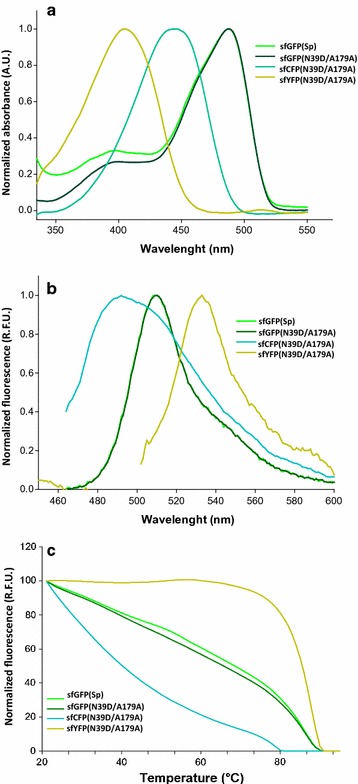
Spectral properties and thermal stability of in vivo-selected sfGFPS102 and its color derivatives compared to the sfGFP(Sp) protein. **a** Absorbance spectra were recorded between 250 and 600 nm using a 1-nm step size in the spectrometer and were normalized to 1 for the respective maximum peaks. A.U., absorbance units. **b** Fluorescence spectra recorded at an excitation wavelength of 485 nm for sfGFPS102, sfGFP(Sp), sfGFPYFPS102 and at 444 nm for sfCFPS102. Curves were normalized to 1 for the respective maximum peak. *R.F.U.* relative fluorescence units. **c** Thermal stability was determined by measuring the emission at 530 nm with an excitation of 488 nm while proteins were subjected to heat in steps of 30 s/1 °C from 20 to 99 °C. *R.F.U.* relative fluorescence units

To gain insight into the reason of the improved performance of sfGFP(N39D/A179A) and its derivatives as compared to the original sfGFP(Sp) protein, we analyzed the thermal in vitro stabilities and the spectral properties (Fig. 7). After amplification with the primer pair LICv1sfGFP_Fs and LICv1sfGFP_Rev, genes were cloned into the expression vector pETHis6TEVLic (1B) and transferred to *E. coli* BL21(DE3) for IPTG-induced expression. After affinity purification and removal of the N-terminal His6-tag, the sfGFP(Sp) and sfGFP(N39D/A179A) proteins showed similar excitation and emission spectra with an absorbance peak at 486 nm and an emission maximum at 510 nm. The cyan variant sfCFPS102 was blue shifted with an absorbance peak at 444 nm and maximum emission at 491 nm. The yellow variant sfYFPS102 showed a bimodal absorbance spectrum corresponding to the protonated and anionic state, with a major peak at 405 nm and a minor peak at 512 nm, while the maximum emission was detected at 524 nm. Remarkably, there was no significant difference in the in vitro thermal stability of sfGFP(N39D/A179A) and its parental protein sfGFP(Sp) (Fig. 7c). While the cyan variant of S102 lost its activity more rapidly during thermal treatment, the introduction of the T203Y mutation in the yellow S102 derivative seemed to stabilize fluorescence emission at higher temperatures.

## Discussion

Gram-positive, thermophilic bacteria of the genera *Parageobacillus* and *Geobacillus* are relevant for biotechnological applications and metabolic engineering for the production of green chemicals from renewable resources such as plant biomass [19, 47]. In this work, we developed a thermostable fluorescent reporter protein, chosen out of several good candidates, that provides a reliable optical readout enabling the quantification of promoter activity changes in single cells or cell populations at 60 °C. We had to overcome two general limitations associated with in vivo functionality of FPs: their strong performance dependence on the host background and temperature dependence of the robustness of folding.

The initial benchmarking of a set of FPs showed that superfolder GFP (sfGFP) proteins were better detectable in *P. thermoglucosidasius* DSM 2542 than the less engineered GFP proteins at 53 °C (Fig. 1a). Given that sfGFP folds an order of magnitude faster than GFP [48], it seems plausible that its folding-accelerating mutations support the functionality at higher temperatures [38]. Since thermal stress of a protein above a certain critical temperature usually leads to improper protein folding and rapid aggregation [49, 50], and can profoundly decrease the maturation efficiency of GFPs [29], it can be speculated that the possibility of occurrence of aggregation-prone or misfolded intermediates is reduced for sfGFPs at elevated temperatures. It has been proposed earlier that faster folding rates within cells allow for greater total fluorescence, because more rapidly folded proteins are protected from degradation and are capable of forming the chromophore [51].

We further observed that sfGFP variants identical at the amino acid level, which were codon-optimized for *G. stearothermophilus* or *B. subtilis*, gave lower fluorescence signals than a variant optimized for *S. pneumoniae* (Fig. 1a and Table 2). This illustrates that the codon usage bias strongly affects the performance of sfGFP variants in *P. thermoglucosidasius*. Similar findings have been reported previously: Although the FP genes were transcribed, the codon usage bias either leads to low levels of translation [52–54], and/or improper folding and loss of functionality, especially at higher temperatures [55]. Thus, codon optimization, a method that introduces favorable and more frequent codons as silent mutations that do not change the primary protein sequence, has been shown to positively influence protein synthesis and lower the rate of mistranslations in cases of some proteins [53, 56, 57]. On the contrary, codon optimization did not yield higher fluorescence or protein expression in other studies [35, 58, 59], indicating that a functional expression depends on a more complex synergy of factors. Although GFP protein levels from a synthetic library of genes that differ randomly at synonymous sites varied 250-fold when expressed in *E. coli*, the codon bias did not correlate with gene expression. GFP levels were rather associated with stability of mRNA folding near the ribosomal binding site, mRNA levels and mRNA degradation patterns [60]. Other factors that might also affect sfGFP expression could be associated with mRNA:ncRNA interactions, tRNA abundance, co-translational folding, the translation rhythm, speed of folding and the macromolecular crowding [61–68].

This underpins that rational engineering by codon optimization algorithms does not always result in improved heterologous gene expression and protein functionality and that in vivo screening approaches might in some cases be better suited to isolate thermostable FP proteins. This is in line with a recently published in vivo selection method called Hot-CoFi, which was demonstrated for successful identification of thermostability-enhancing mutations in a set of proteins [69]. Similarly, our in vivo screen after random mutagenesis of sfGFP(Sp) allowed a direct selection of heat-stable sfGFP variants by instantly omitting mutants with compromised activity (Fig. 2 and Additional file 4: Figure S2).

In general, the amino acid replacements enhanced the hydrophobicity of the proteins by introducing larger hydrophobic residues and exchanged positive or negative charges with polar uncharged or hydrophobic amino acids, respectively. This is in agreement with studies that addressed the differences between mesophilic and thermophilic protein compositions, showing that the hydrophobic content of amino acids is higher in thermophilic proteins [70, 71]. Additionally, a lower overall hydrophobicity leads to diminished tendency to aggregate, as has been shown for GFPuv [72]. We, furthermore, observed an enrichment of mutations in the N terminus and the first beta-strand of sfGFP as well as in the flexible loops and linker regions that connect the β-strands (Fig. 2). Interestingly, similar hotspots, in which stabilizing mutations are clustered in particular regions of proteins, have also been reported to occur in an in vitro screen for enhanced thermostable proteins [69]. The thermostabilizing effect of mutations located on turns of n-caps of helices has been attributed to the reduction of conformational entropy thus enhancing entropic stabilization at higher temperatures [73, 74]. These factors might explain the in vivo FACS selection of these specific mutants; however, much more detailed studies are necessary to judge the impact on folding and maturation of sfGFP of the specific amino acid substitutions and are beyond the scope of this study.

The N39D mutation and the replacement of slow- to fast-translated codons in A179A and H231H (Additional file 5) occurred with higher frequency in the in vivo-isolated proteins, thus indicating that these are indeed crucial substitutions affecting the fluorescence readouts. Especially the combination of N39D and A179A in the mutant sfGFP(N39D/A179A) provided the greatest improvement in in vivo functionality in the DSM 2542 background grown at 60 °C: small signal amplitude with respect to cell-to-cell variation and comparably high fluorescence intensity, which was 885-fold increased in contrast to the original sfGFP(Sp) protein at 60 °C (Figs. 3, 4). However, neither the excitation and emission spectra nor the in vitro temperature stability of sfGFP(N39D/A179A) was altered in comparison to the sfGFP(Sp) protein (Fig. 7). Furthermore, the recombination of all three frequent mutations in the sfGFP(N39D/A179A/H231H) mutant protein impaired its thermal in vivo performance (Fig. 4). This is in good agreement with the findings from the GFP benchmarking (Fig. 1a), indicating that the nucleotide sequence and the codon usage is of high importance for the performance of reporter proteins in this host.

The findings further strongly suggest that a combination of an altered tertiary structure (N39D) and altered translation speed due to a different codon usage frequency (A179A) causes an increase in fluorescence at 60 °C of the sfGFP(N39D/A179A) mutant. In line with this, it has been concluded earlier that differences in the amino acid composition are not the only thermostability modulating factors and that synonymous mutations impact the translation speed of proteins [73]. Additionally, it had been shown that an extrinsic selective force for gene mutations in thermophiles was particularly linked to the process of synonymous codon usage for arginine and isoleucine [71]. It is a generally accepted view that silent substitutions alter the translation elongation rates and efficiencies of protein folding [75–78]. Thus, in the case of the A179A mutation, the replacement of a slow (rare) with a fast (frequent) arginine codon might enhance the translation speed of the protein variant and accelerate its maturation, which might confer protection against degradation of misfolded or incompletely folded intermediates. Interestingly, the N39D mutation removed asparagine, which is considered a thermolabile amino acid [73]. Moreover, the N39D replacement reversed the original superfolder mutation Y39N, which was shown to initiate the formation of an alpha-helix at the loop between the second and third beta-strands of the barrel and contributed to the folding robustness by stabilizing this protein region [38]. It would thus be interesting to perform crystallization studies of sfGFP(N39D/A179A), to shed light on the contribution of 39D to the in vivo thermostability of the protein. Thus, current studies are underway to determine whether the translation rate, mRNA or protein decay contribute to the improved in vivo functionality of sfGFP(N39D/A179A) in *P. thermoglucosidasius*.

Based on previously reported mutations shifting the spectral properties of GFP [45, 46], we constructed cyan and yellow color derivatives of sfGFP(N39D/A179A). These variants showed the expected modifications in excitation and emission spectra in vitro (Fig. 7a, b) and could be detected in in vivo expression experiments in *P. thermoglucosidasius* (Fig. 6). To our knowledge, this is the first report of sfCFP and sfYFP proteins being successfully expressed in thermophilic spore-forming bacteria in vivo. Both variants displayed altered thermostability profiles compared to the sfGFP(N39D/A179A) protein (Fig. 7c), indicating that the introduced amino acid substitutions impact the in vitro unfolding kinetics. This is presumably based on noncovalent interactions of the altered chromophore region with the β-barrel that influence the protein stability [30], and on differences in vibrational disruption of the local fluorophore environment [79], which has been reported to be much more stable due to extended pi–pi stacking interactions of 203Y with the chromophore in the yellow exciting FP [46].

Since the in vivo-applicability of sfGFP(N39D/A179A), sfCFP(N39D/A179A) and sfYFP(N39D/A179A) was proven in a set of additional industrially relevant (moderately) thermophilic bacteria (Figs. 5, 6), we expect that this set of proteins has numerous applications for industrial, as well as basic research questions in these organisms. For instance, it might be possible to use sfGFP(N39D/A179A) as a folding reporter, similar to the original sfGFP, to screen for correctly folded proteins such as biotechnologically relevant enzymes when expressed at high temperatures.

## Conclusions

We demonstrated that a combination of random undirected mutagenesis of the GFP gene with in vivo isolation of the best performing clones by fluorescence-assisted cell sorting turned out to be a powerful strategy to isolate well-expressed variants with significantly improved thermostability in thermophilic bacteria.

We present a mutant of sfGFP, termed sfGFP(N39D/A179A), which is 885-fold brighter than the benchmarked parental protein sfGFP(Sp) and active *in P. thermoglucosidasius* as well as in *B. smithii, B. coagulans, B. methanolicus* and *G. thermodenitrificans* grown between 45 and 65 °C. This demonstrates its broad applicability in diverse thermophilic bacteria of biotechnological relevance. sfGFP(N39D/A179A) and its cyan and yellow color derivatives are thus of interest for a variety of applications in metabolic and cellular engineering. In contrast to previously published sfGFP or GFP variants, whose thermostability or functionality in *P. thermoglucosidasius* was either not proven in vivo or not assessed at the single-cell level, sfGFP(N39D/A179A) shows a comparably narrow signal amplitude and an extreme brightness in single cells. This is important to perform meaningful quantification of weak, strong, and inducible promoter activity changes at the single cell and at the population level.

The novel variants can be applied for the determination of promoter and RBS strengths, to monitor metabolite conversions or fluxes and for in situ localization of proteins in the cellular environment. Therefore, it is now possible to perform real-time monitoring of biological processes in thermophilic bacteria that are of industrial and biotechnological interest.

## Additional files

**Additional file 1.** Oligonucleotides designed and used in this study.

**Additional file 2.** Plasmids used in this study.

**Additional file 3.** Plasmid map of pNW-P_pta_-GFPx-3TER and examples of expression kinetics of different GFP types in *P. thermoglucosidasius*.

**Additional file 4.** Workflow for in vivo enrichment of thermostable sfGFP mutants and single variant screening in *P. thermoglucosidasius*.

**Additional file 5.** Table listing examples of thermostable sfGFP variants isolated from FACS enrichment in *P. thermoglucosidasius* DSM 2542 at 60 °C.

## Abbreviations

FP: fluorescent protein
GFP: green fluorescent protein
sfGFP: superfolder green fluorescent protein
CFP: cyan fluorescent protein
YFP: yellow fluorescent protein
ex: excitation
em: emission
