# Supplementary material for: In vivo selection of sfGFP variants with improved and reliable functionality in industrially important thermophilic bacteria
Source: Biotechnol Biofuels. 2018 Jan 17;11:8. doi: 10.1186/s13068-017-1008-5 (PMC5771013; doi:10.1186/s13068-017-1008-5)
Supplement: Supplementary file 1 — Additional file 1. Oligonucleotides designed and used in this study. [file 13068_2017_1008_MOESM1_ESM.docx]

**Additional file 1**

**Table S1. Oligonucleotides designed and used in this study**

| **Primer name** | **Sequence (5’ to 3’)** | **Purpose** |
| --- | --- | --- |
| pNW33N_for | GGTGCGTTGAAGTGTTGGTATG | Sequencing primer for MCS inserts in pNW33N and colony PCR |
| pNW33N_rev | TCTAATGTCACTAGGGCTCGC | Sequencing primer for MCS inserts in pNW33N and colony PCR |
| 3TER_for | TGAGCATGCTAGGACGCCGCCAAGCCAGCT | Amplification of 3-fold terminator structure from pKB01-sfGFP(Sp) |
| 3TER_rev | AGAAAGCTTGAATCTTGCTTGGCAAGGTTC | Amplification of 3-fold terminator structure from pKB01-sfGFP(Sp) |
| PptaGeoS_for | GCAGAATTCAACGCTCGAATGCCGCATGTA | Amplification of constitutive *pta* promoter from *P. thermoglucosidasius* DSM 2542 |
| PptaGeo_rev | GATAGACCCGGGGAACGAATCCTCCCTAATGTT | Amplification of constitutive *pta* promoter from *P. thermoglucosidasius* DSM 2542 |
| GFPmut3A_for | GGATCTAGAATGAGTAAAGGAGAAGAACT | Amplification of *gfpmut3A* from pAD123 |
| GFPmut3A_rev | AGTGCATGCTTATTTGTATAGTTCATCCA | Amplification of *gfpmut3A* from pAD123 |
| GFPuv_for | GGATCTAGAATGAGTAAAGGAGAAGAA | Amplification of *gfpuv* from pSG1156 |
| GFPuv_rev | ACGGCATGCTTATTTGTAGAGCTCATCCA | Amplification of *gfpuv* from pSG1156 |
| sfGFP_Gst_F | AACTCTAGAATGGCCATGGGCTCCAAAGGC | Amplification of *sfgfp*Gst from plasmid PRHIII-sfGFP-pNW33N |
| sfGFP_Gst_R | ATAGCATGCCGGCCGCTACTAGTATTACTT | Amplification of *sfgfpGst* from plasmid PRHIII-sfGFP-pNW33N |
| pKB01_FP_for | CCGCTCTAGATGATTAACTAATAAGGAGGACAAAC | Amplification of FP genes from pKB01 series |
| pKB01_FP_rev | CCAGCATGCAAAGAATCTTGCTTGGCAAGGTTC | Amplification of FP genes from pKB01 series |
| pKB01derMut_F | GCGTCTAGAACTAATAAGGAGGACAAACATG | Amplification primer for random mutagenesis of sfGFP(Sp) from pKB01_sfgfp(Sp) plasmid |
| pKB01derMut_R | CCTGCATGCCCTTGACTAGTGCTCATTATTA | Amplification primer for random mutagenesis of sfGFP(Sp) from pKB01_sfgfp(Sp) plasmid |
| sfGFP_H231H_f | GTTACAGCAGCAGGTATCACTCATGGTATGGACGAA | Site-directed mutagenesis for H231H introduction in sfGFP(N39D/A179A) |
| sfGFP_H231H_r | TTCGTCCATACCATGAGTGATACCTGCTGCTGTAAC | Site-directed mutagenesis for H231H introduction in sfGFP(N39D/A179A) |
| sfGFP_Y66W_R | AACATTGTACTCCCCATGTCAATGTTGTA | Site-directed mutagenesis of  sfGFP(N39D/A179A) for exchange Y66W |
| sfGFP_Y66W_F | TACAACATTGACATGGGGAGTACAATGTT | Site-directed mutagenesis of  sfGFP(N39D/A179A) for exchange Y66W |
| sfGFP_T203Y_R | GAAAGAACTGATTGGTAAGAAAGGTAGTGG | Site-directed mutagenesis of  sfGFP(N39D/A179A) for exchange T203Y |
| sfGFP_T203Y_F | CCACTACCTTTCTTACCAATCAGTTCTTTC | Site-directed mutagenesis of  sfGFP(N39D/A179A) for exchange T203Y |
| sfGFP_Xba_F | GTGTCTAGAATGTCAAAAGGAGAAGAACTTT | Site-directed mutagenesis of  sfGFP(N39D/A179A) for exchange T203Y or Y66W |
| sfGFP_Sph_R | ATTGCATGCCCTTGACTAGTGCTCATTATTA | Site-directed mutagenesis of  sfGFP(N39D/A179A) for exchange T203Y or Y66W |
| LICv1sfGFP_Fs | TACTTCCAATCCAATGCATCAAAAGGAGAAGAACTTTTTACA | Amplification of FPs from pNW-P_pta_-sfGFP(N39D/A179A), pNW-P_pta_-sfCFPS102 and pNW-P_pta_-sfYFPS102 |
| LICv1sfGFP_Rs | TTATCCACTTCCAATGTTATTATTTATAAAGTTCGTCCATAC | Amplification of FPs from pNW-P_pta_-sfGFP(Sp), pNW-P_pta_-sfGFP(N39D/A179A), pNW-P_pta_-sfCFPS102 and pNW-P_pta_-sfYFPS102 |
| LicV1sfGFP_F | TACTTCCAATCCAATGCATCAAAAGGAGAAGAACTTTTTACAGGT | Sequencing primer for pETHis6TEVLic (1B) plasmid |
| LicV1sfGFP_R | TTATCCACTTCCAATGTTATTATTTATAAAGTTCGTCCATACCGT | Sequencing primer for pETHis6TEVLic (1B) plasmid |
