# Supplementary material for: In vivo selection of sfGFP variants with improved and reliable functionality in industrially important thermophilic bacteria
Source: Biotechnol Biofuels. 2018 Jan 17;11:8. doi: 10.1186/s13068-017-1008-5 (PMC5771013; doi:10.1186/s13068-017-1008-5)
Supplement: Supplementary file 2 — Additional file 2. Plasmids used in this study. [file 13068_2017_1008_MOESM2_ESM.docx]

**Additional file 2**

**Table S2. Plasmids used in this study**

| **Plasmids** | **Relevant characteristics** | **Reference/source** |
| --- | --- | --- |
| **Plasmids used as templates for FP subcloning** | | |
| pAD123 | Amp^r^ Kan^r^ P_upp_-*gfpmut3A* | [[81](#_ENREF_81)] |
| pSG1156 | Amp^r^ Cm^r^ *gfp(uv)* | [[81](#_ENREF_81)] |
| pKB01_gfp+ | Amp^r^ Tet^r^ P_Zn_-*gfp+* | [[35](#_ENREF_35)] |
| pKB01_gfp(Sp) | Amp^r^ Tet^r^ P_Zn_-*gfp*(*Sp*) | [[35](#_ENREF_35)] |
| pKB01_sfgfp(Bs) | Amp^r^ Tet^r^ P_Zn_-*sfgfp*(*Bs*) | [[35](#_ENREF_35)] |
| pKB01_sfgfp(Sp) | Amp^r^ Tet^r^ P_Zn_-*sfgfp*(*Sp*) | [[35](#_ENREF_35)] |
| pKB01_sfgfp(iGEM) | Amp^r^ Tet^r^ P_Zn_-*sfgfp*(*iGEM*) | [[35](#_ENREF_35)] |
| PRHIII-sfGFP-pNW33N | Cm^r^ P_RHIII_-*sfGFP(Gst)* | [[40](#_ENREF_40)] |
| ***E. coli-Bacillus* shuttle vectors constructed for FP expression** | | |
| pNW33N | *E. coli- Bacillus* shuttle vector, Cm^r^, pTHT15 origin of replication, rolling-circle (RC) mechanism of replication | BSGC |
| pNW-P_pta_-3TER | Derivative of pNW33N, containing constitutive promoter of *P. thermoglucosidasius* DSM 2542 phosphate acetyltransferase (*pta*) gene and three transcriptional terminators derived from plasmid pKB01-sfGFP(Sp) | This study |
| pNW-GFPmut3A | Derivative of pNW-P_pta_-3TER containing *gfpmut3A* | This study |
| pNW-GFPuv | Derivative of pNW-P_pta_-3TER containing *gfpuv* | This study |
| pNW-GFP+ | Derivative of pNW-P_pta_-3TER containing *gfp+* | This study |
| pNW-GFP(Sp) | Derivative of pNW-P_pta_-3TER containing *gfp(Sp)* | This study |
| pNW-sfGFP(Bs) | Derivative of pNW-P_pta_-3TER containing *sfGFP(Bs)* | This study |
| pNW-sfGFP(Sp) | Derivative of pNW-P_pta_-3TER containing *sfGFP(Sp)* | This study |
| pNW-sfGFP(Gst) | Derivative of pNW-P_pta_-3TER containing *sfGFP(Gst)* | This study |
| pNW-sfGFP(iGEM) | Derivative of pNW-P_pta_-3TER containing *sfGFP(iGEM)* | This study |
| pNW-sfGFP(N39D/A179A) | Derivative of pNW-P_pta_-3TER encoding thermostable sfGFP variant sfGFP(N39D/A179A) | This study |
| pNW-sfGFP(N39D/A179A/H231H) | Derivative of pNW-P_pta_-sfGFP(N39D/A179A)-3TER with additional H231H silent mutation | This study |
| pNW-sfCFP(N39D/A179A) | Cyan color derivative (Y66W) of pNW-sfGFP(N39D/A179A) | This study |
| pNW-sfYFP(N39D/A179A) | Yellow color derivative (T203Y)of pNW-sfGFP(N39D/ASeite **1** von **2**179A) | This study |
| **Plasmids used for recombinant expression and purification of proteins from *E.coli*** | | |
| pETHis6TEVLic (1B) | Kan^R^, N-terminal His_6_-tag and TEV protease cleavage site for His_6_ removal, IPTG-inducible protein expression | pET His6 TEV LIC cloning vector (1B) was a gift from Scott Gradia (Addgene plasmid # 29653) |
| pETHisTEV-sfGFP(Sp) | Derivative of pETHis6TEVLic containing *sfGFP(Sp)* | This study |
| pETHisTEV-sfGFP(N39D/A179A) | Derivative of pETHis6TEVLic containing *sfGFP(N39D/A179A)* | This study |
| pETHisTEV-sfCFPS102 | Derivative of pETHis6TEVLic containing *sfCFP*(N39D/A179A) | This study |
| pETHisTEV-sfYFP(N39D/A179A) | Derivative of pETHis6TEVLic containing *sfYFP*(N39D/A179A) | This study |

BSGC, Bacillus Genetic Stock Centre; Cm^r^, chloramphenicol resistance cassette; Amp^r^, Ampicillin resistance cassette, Kan^r^, Kanamycin resistance cassette, Tet^r^, tetracyclin resistance cassette
