# Supplementary material for: In vivo selection of sfGFP variants with improved and reliable functionality in industrially important thermophilic bacteria
Source: Biotechnol Biofuels. 2018 Jan 17;11:8. doi: 10.1186/s13068-017-1008-5 (PMC5771013; doi:10.1186/s13068-017-1008-5)
Supplement: Supplementary file 3 — Additional file 3. Plasmid map of pNW-Ppta-GFPx-3TER and examples of expression kinetics of different GFP types in P. thermoglucosidasius. [file 13068_2017_1008_MOESM3_ESM.docx]

**Additional file 3**

**Figure S1. Kinetics of GFP expression in *P. thermoglucosidasius* DSM 2542.** (A) pNW33N-derived GFP expression vector including the constitutive promoter of the phosphate acetyltransferase gene (*pta*) from *P. thermoglucosidasius*, a three-fold transcription terminator (3TER) derived from plasmid pKB01 and the XbaI/SphI insertion site for GFP variants (GFPx). Flow cytometry analysis of fluorescence intensities of transformed *P. thermoglucosidasius* grown for 2 to 12 hours at 53°C in TGP broth in comparison to the auto-fluorescence of the wild-type strain (blue vertical bar) (B) example of a conventional GFP (GFP+) (C) example of a superfolder GFP codon optimized for *Streptococcus pneumoniae* (sfGFP(Sp)) and (D) example of a superfolder GFP codon optimized for *Geobacillus stearothermophilus* (sfGFP(Gst)).
