# Supplementary material for: In vivo selection of sfGFP variants with improved and reliable functionality in industrially important thermophilic bacteria
Source: Biotechnol Biofuels. 2018 Jan 17;11:8. doi: 10.1186/s13068-017-1008-5 (PMC5771013; doi:10.1186/s13068-017-1008-5)
Supplement: Supplementary file 4 — Additional file 4. Workflow for in vivo enrichment of thermostable sfGFP mutants and single variant screening in P. thermoglucosidasius. [file 13068_2017_1008_MOESM4_ESM.docx]

**Additional file 4**

**Figure S2. Workflow for *in vivo* enrichment of thermostable sfGFP(Sp) mutants and single variant screening in *P. thermoglucosidasius* DSM 2542.** (A) A library of ca. 45,000 sfGFP(Sp) mutants was transformed into DSM 2542. Cells were inoculated from a pre-culture into 50 ml of TGP broth and grown at 55°C, 60°C or 65°C until mid-exponential growth phase. Ca. 100,000 of the mutants with highest sfGFP emission were isolated by FACS and further plated on TGP broth and re-inoculated in a second culture for subsequent enrichment of cells showing a high fluorescence at 60°C or 65°C, respectively. Subsequent enrichment by FACS was performed four times. (B) Example of *in vivo* enrichment: The upper panel shows the distribution of fluorescence in single cells of the DSM 2542 library grown at 55°C after the first round of FACS. The red bar indicates the mean fluorescence intensity. The lower panel shows the same library after successful second round of enrichment. (C) Single colonies obtained by FACS were restreaked and their fluorescence intensity was assessed at the mid-exponential growth phase by flow cytometry. Fifty of the most thermostable mutants were sequenced to analyze the underlying mutations in the sfGFP gene.
