# Supplementary material for: In vivo selection of sfGFP variants with improved and reliable functionality in industrially important thermophilic bacteria
Source: Biotechnol Biofuels. 2018 Jan 17;11:8. doi: 10.1186/s13068-017-1008-5 (PMC5771013; doi:10.1186/s13068-017-1008-5)
Supplement: Supplementary file 5 — Additional file 5. Table listing examples of thermostable sfGFP variants isolated from FACS enrichment in P. thermoglucosidasius DSM 2542 at 60 °C. [file 13068_2017_1008_MOESM5_ESM.docx]

**Additional file 5**

**Table S3. Example of sfGFP mutants isolated from FACS enrichment in *P. thermoglucosidasius* DSM 2542 at 60°C**

| **Clone number** | **(Silent) amino acid replacements compared to the original sfGFP(Sp) protein** | **AA substitutions (functional)** | | **Codon frequency change compared to original wt sfGFP(Sp) (ratio=codon usage mutant/codon usage wt)** |
| --- | --- | --- | --- | --- |
| 1 | H231H | H231H | Synonymous | 3.11 |
| 2 | N39D, A179A | N39D | Polar uncharged-> negatively charged | 0.95 |
|  |  | A179A | Synonymous | 1.19 |
| 3 | D19N, K214N | D19N | Negatively charged->polar uncharged | 0.66 |
|  |  | K214N | Positively charged-> polar uncharged | 0.32 |
| 4 | G4G, T9P, I14F, V16G, N39D, T225A | G4G | Synonymous | 0.62 |
|  |  | T9P | Polar uncharged->nonpolar | 0.61 |
|  |  | I14F | Hydrophobic, aliphatic->hydrophobic, aromatic | 1.37 |
|  |  | V16G | Hydrophobic->nonpolar | 0.46 |
|  |  | N39D | Polar uncharged-> negatively charged | 0.95 |
|  |  | T225A | Polar uncharged->hydrophobic | 1.13 |
| 5 | L7F, N39D | L7F | Hydrophobic->hydrophobic (large) | 1.61 |
|  |  | N39D | Polar uncharged-> negatively charged | 0.95 |
| 6 | L15W, F84L, H231H | L15W | Hydrophobic->hydrophobic (large) | 0.47 |
|  |  | F84L | Hydrophobic->hydrophobic (small) | 0.76 |
|  |  | H231H | Synonymous | 3.11 |
| 7 | K131R, H231H | K131R | Positively charged->positively charged | 0.11 |
|  |  | H231H | Synonymous | 3.11 |
| 8 | N39K, K101N, M218L | N39K | Polar uncharged-> positively charged | 3.14 |
|  |  | K101N | Positively charged->polar uncharged | 0.39 |
|  |  | M218L | Hydrophobic->hydrophobic (smaller) | 0.89 |
| 9 | T9P, V12I, K85I | T9P | Polar uncharged->nonpolar | 0.61 |
|  |  | V12I | Hydrophobic->hydrophobic (large) | 0.75 |
|  |  | K85I | Positively charged->hydrophobic, aliphatic | 0.17 |
| 10 | T50T, K101N, A179A | T50T | Synonymous | 1.29 |
|  |  | K101N | Positively charged->polar uncharged | 0.39 |
|  |  | A179A | Synonymous | 1.19 |
